# Island Latissimus Dorsi Muscle Flap and a Perforator Flap in Repairing Post-Gunshot Thoracic Spine CSF Fistula: Case Presentation

**DOI:** 10.1155/2015/219535

**Published:** 2015-09-28

**Authors:** Nangole F. Wanjala, Khainga Ominde Stanley

**Affiliations:** Department of Surgery (Plastic), University of Nairobi, P.O. Box 30197, Nairobi 00100, Kenya

## Abstract

Persistent posttraumatic CSF fluid leakage may present a challenge to manage. Failure to address the leakage may result in complications such as meningitis, septicemia, radiculopathy, muscle weakness, and back pains. While the majority of the leakages may be managed conservatively, large dura defects as a result of gunshot wounds or motor vehicle accidents are best managed by surgical interventions. This may range from primary closure of the defect to fascial grafts, adhesive glues, and flaps. We present our experience with the use of flaps in a patient who had sustained such wounds in the thoracic spine. An island latissimus dorsal flap and a perforator fasciocutaneous flap were used to close the defect. Postoperatively the patient recovered well and the wounds healed without any complications.

## 1. Introduction

Posttraumatic cerebrospinal fluid fistula may result in bacterial translocation into the subarachnoid space with subsequent meningitis that could be life-threatening. Other complications associated with this include backaches, muscle weaknesses, and radiculopathy. Iatrogenic leakages could be managed conservatively with good outcome [1, 2]. This clinically is noticed by the surgeon as clear cerebrospinal fluid along the incision line or fluctuant swelling at the surgical site with no other complications. The conservative management could include bed rest, pressure dressings, and diversionary lumbar drainage. However leakages as a result of gunshots are unlikely to conceal on there on and would thus require surgical intervention. Traditionally the surgical intervention requires the reconstruction of the dura mater with either tensor fascia lata graft or the temporalis fascia [3–5]. Due to the need to adequately expose the remaining dura mater, so as to allow for an airtight closure of the dura, laminectomy may have to be done to improve on access.

In this presentation we share our experience with a patient whom we managed with an island latissimus dorsi muscle and a perforator flap, thus obviating the need for the use of fascial graft and laminectomy.

## 2. Case Presentation

**Figure 1:**
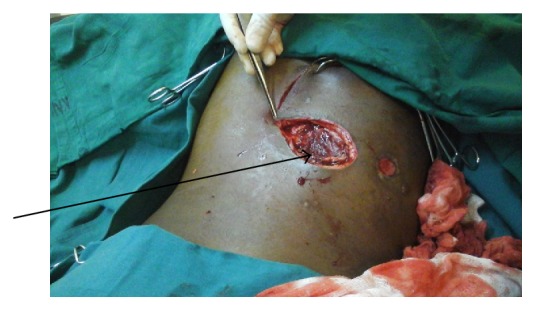
Gunshot wound with arrow pointing at the bullet exit.

A 21-year-old girl presented to us with history of a gunshot wound to the thoracic spine as a result of an attack by an extreme militia group in her school. She subsequently developed paraplegia with a wound at the T6 and T7 spine level measuring about 5 cm in diameter (Figure 1). The wound was managed with serial surgical debridement. Tissue taken for culture and sensitivity grew* Escherichia coli* sensitive to cefuroxime among other antibiotics. She was thus put on intravenous cefuroxime. Dressings were done with antimicrobial foam dressing. After about three weeks of the above management her wound had improved. She however persistently leaked cerebrospinal fluid through the dressing. A decision was thus made to have the wound closed surgically.

**Figure 2:**
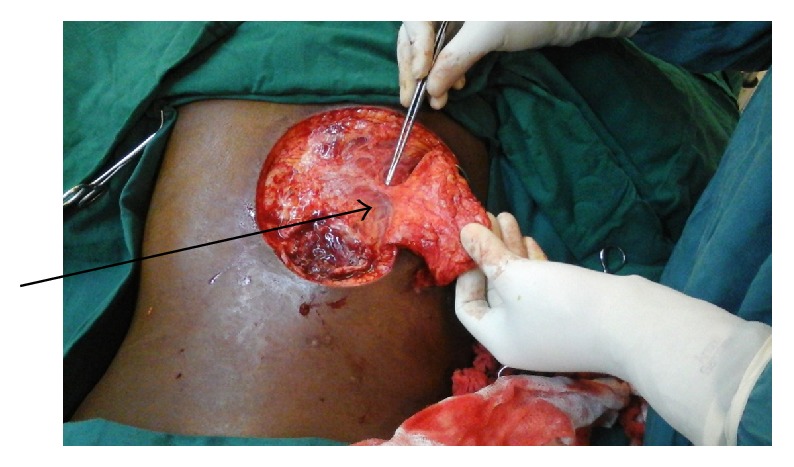
Perforator fasciocutaneous flap with arrow pointing to the blood vessels.

**Figure 3:**
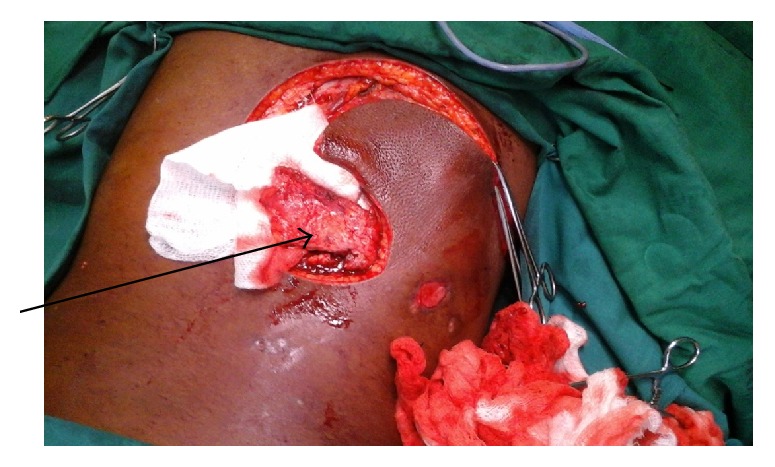
Island latissimus dorsi segmental muscle flap.

**Figure 4:**
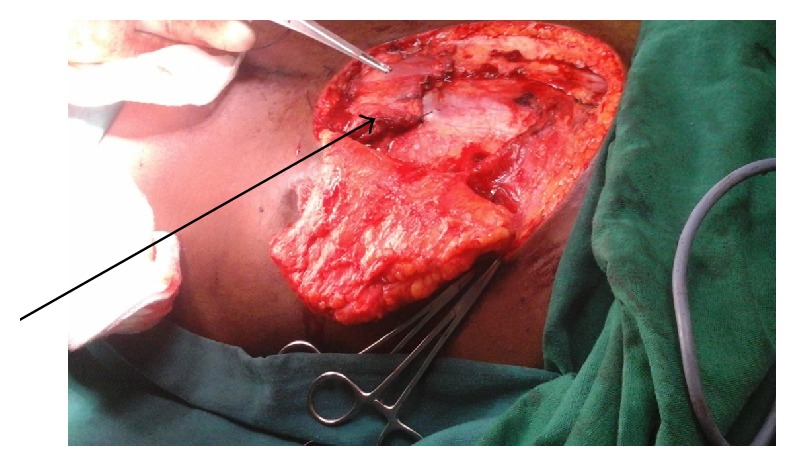
Latissimus dorsi muscle inserted into the defect.

**Figure 5:**
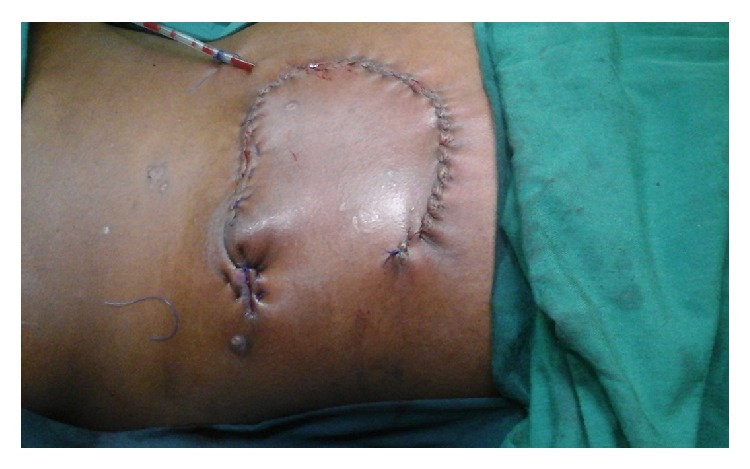
Wound completely covered with the fasciocutaneous flap with a drain in situ.

**Figure 6:**
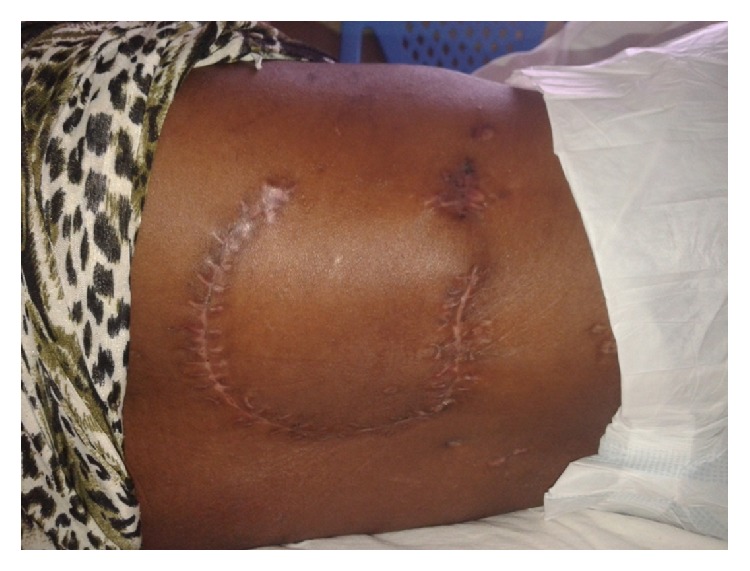
Wound fully healed 2 weeks after surgery.

Operatively, a repeat tissue debridement was done and all nonviable tissues and debris were removed. With the aid of a hand held Doppler a suitable perforator was identified in the close proximity of the wound. A fasciocutaneous flap was then fashioned on this perforator (Figure 2). The latissimus dorsi muscle was then identified. A segment of the muscle was then raised as an island flap based on one of the vessels through the paraspinous muscle (Figure 3). The muscle flap was then mobilized and used to fill the dura and the muscle defect over the spine in two layers (Figure 4). The perforator fasciocutaneous flap was then advanced over the wound and closed similarly in two layers with sutures. A closed drain was put between the two flaps (Figure 5). The drain was removed on the 10th postoperative day once it was noted to be inactive. There were no complications and the wounds had healed completely at about two weeks after surgery (Figure 6).

## 3. Discussions

Cerebrospinal fluid leakage is a potentially dangerous condition that could be detrimental to the patient's life. Inappropriate management of this condition could result in meningitis, paraspinal abscess, brain abscess, and septicemia that could lead to death. Other complications include backaches, radiculopathy, and muscle weaknesses.

The majority of the CSF leakages in the lumbar and thoracic region are iatrogenic in nature [1]. They do occur as a result of surgery to the spine with tears to the meninges. Most of these tears are small resulting in minor leakages that could be managed conservatively with good outcome [2, 3]. Conservative management entails bed rest, pressure dressings, and lumbar cerebrospinal fluid drainage procedures. Large tears either iatrogenic or otherwise would result in excess leakage of cerebrospinal fluids and are unlikely to heal conservatively. Surgical management of these leakages includes direct wound closure, use of adhesive glues, fascial lata grafts, muscle grafts, and fat grafts [2–7].

CSF leakages as a result of gunshots on the other hand provide extra challenges. There is usually extensive soft tissue loss. The injury may be associated with fractures of the vertebral bones that may need to be stabilised. The defects in the dura are likely to be wide either due to the tract of the bullet or as a result of the bone fragments blasted away with the bullet.

It may thus be difficult to manage such injuries with the conventional management strategies as mentioned above. Effective management of such defect will demand addressing the extensive soft tissue loss as well as providing well-vascularised tissue that can integrate with the surrounding tissues and thus assist in closing the fistula.

In our patient this was achieved by using a segment of the latissimus dorsi muscle as well as a perforator fasciocutaneous flap. The muscle was mobilized on one of the paravertebral blood vessels as an island flap. Due to its bulk it was able to fill the gunshot wound adequately, completely obliterating any dead space and also acting as a biological barrier to the continuous leakage of the CSF. Further theoretical advantages of the muscle included drainage of the CSF back to the circulation through its rich capillary network. The fasciocutaneous flap on the other hand was able to provide adequate skin and fascia over the wound, allowing for good soft tissue padding over the muscle. It also enabled the defect to be covered with tissues away from the zone of injury with better vascularity and minimal chances of contamination.

In conclusion island latissimus dorsi muscle and a perforator flap are alternatives in covering open wound defects over the lower thoracic spine region with cerebrospinal fluid fistula. The flaps are relatively easy to raise and have a reach vascularity that allows them to readily integrate with the tissues at the recipient site.
